# Apparent Cerebellar Volumetric Stabilization and Emergent Cortical Hyperexcitability During Long-Acting Injectable Aripiprazole Maintenance in Adolescent Bipolar Disorder

**DOI:** 10.3390/pediatric18040114

**Published:** 2026-08-14

**Authors:** Erasmia I. Koiliari, Christos Tsitsipanis, Marianna Evangelia Kapsetaki, Emmanouil L. Pasparakis

**Affiliations:** 1 Regional Mental Health Services Network of Crete, 71500 Heraklion, Greece; 2 Laboratory of Alcohology, Department of Internal Medicine, Medical School, University of Crete, 70013 Heraklion, Greece; 3 General Hospital of Agios Nikolaos of Crete, 72100 Agios Nikolaos, Greece; 4 Neurosurgery Department, University Hospital of Heraklion, 70013 Heraklion, Greece; 5 School of Medicine, University of Crete, 70013 Heraklion, Greece

**Keywords:** bipolar disorder, adolescent, cerebellar atrophy, vermis, aripiprazole long-acting injectable, seizure threshold, myoclonus, EEG

## Abstract

Background and Clinical Significance: This report investigates the complex intersection of macrostructural neuroprotection and cortical hyperexcitability during long-acting atypical antipsychotic maintenance. We present a novel clinical case demonstrating an apparent absence of detectable cerebellar vermis atrophy progression during long-acting injectable (LAI) aripiprazole maintenance, which temporally coincided with the emergence of a potential epileptogenic risk in an adolescent with bipolar disorder (BD) and cannabis use disorder. Beyond motor precision, the vermis modulates emotional-cognitive networks; deficits in these circuits impair impulse control, frequently driving comorbid substance use in youth. Case Presentation: A 16-year-old female with BD and cannabis use disorder presented with pronounced cerebellar and vermis atrophy on brain CT during an acute behavioral crisis. Following diagnostic reformulation, maintenance therapy was initiated via off-label monthly LAI aripiprazole (400 mg) alongside lithium. At 9-month follow-up, psychiatric relapses and cannabis use remitted completely. Repeat CT suggested macrostructural stability with zero apparent atrophy progression. However, during the 9th month, she abruptly developed daily generalized myoclonus. An awake electroencephalogram (EEG) revealed intense cortical hyperexcitability, documenting frequent interictal and ictal epileptiform discharges with generalized 3–4 Hz spike-wave complexes synchronized with the clinical myoclonus. Introduction of levetiracetam (500 mg BID) and cessation of adjunct methylphenidate led to complete clinical and neurophysiological remission. Conclusions: LAI aripiprazole may favorably influence the macrostructural trajectory of the cerebellum/vermis in adolescent BD, suggesting volume stabilization. However, clinicians must monitor for a drug-induced lowering of the seizure threshold, where macrostructural volume preservation coexists with microstructural electrical destabilization.

## 1. Introduction

Bipolar disorder (BD) is a complex neuropsychiatric condition characterized by recurrent episodes of mania and depression, fundamentally disrupting behavior, cognition, and affective regulation [1]. Although historically conceptualized primarily as a fronto-limbic dysregulation, contemporary neuroimaging increasingly implicates the cerebellum in the pathophysiology of mood disorders [2,3]. The functional topography of the cerebellum demonstrates a clear division: while the anterior lobe coordinates motor output, the posterior lobe and the midline vermis are heavily involved in cognitive processing and emotional regulation [2].

The involvement of the cerebellum in affective homeostasis aligns with clinical hypotheses framing this structure as an “emotional pacemaker” [4,5]. Recent neuroanatomical evidence has solidified this concept by identifying reciprocal connections between the cerebellar vermis and limbic structures—specifically the nucleus accumbens and the prefrontal cortex—constituting the so-called “limbic cerebellum” [6,7]. Through these loops, the vermis provides cognitive and emotional fine-tuning necessary for executive decision-making [7,8].

Structural neuroimaging studies demonstrate that the volume of the cerebellar vermis is highly sensitive to the course of BD [9,10]. In morphometric analyses, the vermis is typically segmented into subregions based on sagittal orientation, where the V2 (superior posterior, lobules VI–VII) and V3 (inferior posterior, lobules VIII–X) areas represent main nodes of affective processing [10]. Multi-episode BD patients often exhibit reduced V2 and V3 vermis volumes compared to first-episode patients, suggesting that cumulative affective episodes accelerate neuroanatomical degradation [9,10].

In adolescents, this structural vulnerability carries profound clinical implications. The developmental trajectory of the cerebellum extends well into late adolescence, rendering it susceptible to severe mood fluctuations, oxidative stress, and neuroinflammation. Disruption of fronto-cerebellar circuitry during this critical window severely impairs impulse control and degrades social decision-making faculties [11]. Clinically, this executive deficit heavily predisposes youth to comorbid substance use disorders, particularly cannabis use disorder, creating a highly refractory dual-diagnosis phenotype characterized by high relapse rates and medication non-compliance.

Consequently, therapeutic strategies in adolescent BD target both symptom stabilization and potential neuroprotection to halt this anatomical regression [12,13]. Second-generation antipsychotics with partial dopamine D2 receptor agonism, such as aripiprazole, have emerged as promising agents that may promote neuroplasticity and preserve structural integrity [14,15,16,17]. However, atypical antipsychotics also possess complex neuroelectrophysiological footprints, occasionally modulating the seizure threshold and unmasking cortical hyperexcitability in susceptible neuroanatomical substrates.

Here, we present the case of an adolescent patient with severe, multi-episode BD and comorbid cannabis use disorder, where the administration of long-acting injectable (LAI) aripiprazole was associated with the structural stabilization of cerebellar and vermis atrophy, but concurrently unmasked diffuse cortical hyperexcitability manifested as generalized myoclonus.

## 2. Case Presentation

### 2.1. Clinical History and Index Presentation

A 16-year-old female adolescent presented involuntarily to the Emergency Department of the University General Hospital of Heraklion (Crete, Greece), a tertiary academic medical center within the regional mental health services network, escorted by police authorities, following a high-acuity behavioral crisis. The patient had an extensive psychiatric history characterized by severe behavioral dysregulation and a total of nine prior psychiatric admissions—the vast majority of which were involuntary—within the preceding two years (ages 14 to 16). Historically, her clinical presentation had been attributed by treating physicians to a severe “Conduct Disorder.”

The patient had been discharged from an inpatient unit just four days prior to this current admission under the same working diagnosis of Conduct Disorder. Her prescribed pharmacological regimen at discharge consisted of lithium carbonate (300 mg twice daily), escitalopram (10 mg, half a tablet daily), and lurasidone (37 mg once daily). Comorbid somatic conditions included primary hypothyroidism, well-managed with a daily maintenance dose of levothyroxine (25 mcg once daily); endocrine evaluations confirmed a euthyroid state. Within the four days following her discharge, the patient eloped from the family home without parental supervision. During this period, she exhibited marked manic impulsivity, hypersexuality, and severe impairment in social judgment, leading to high-risk behaviors that required immediate legal and parental intervention.

**Figure 1:**
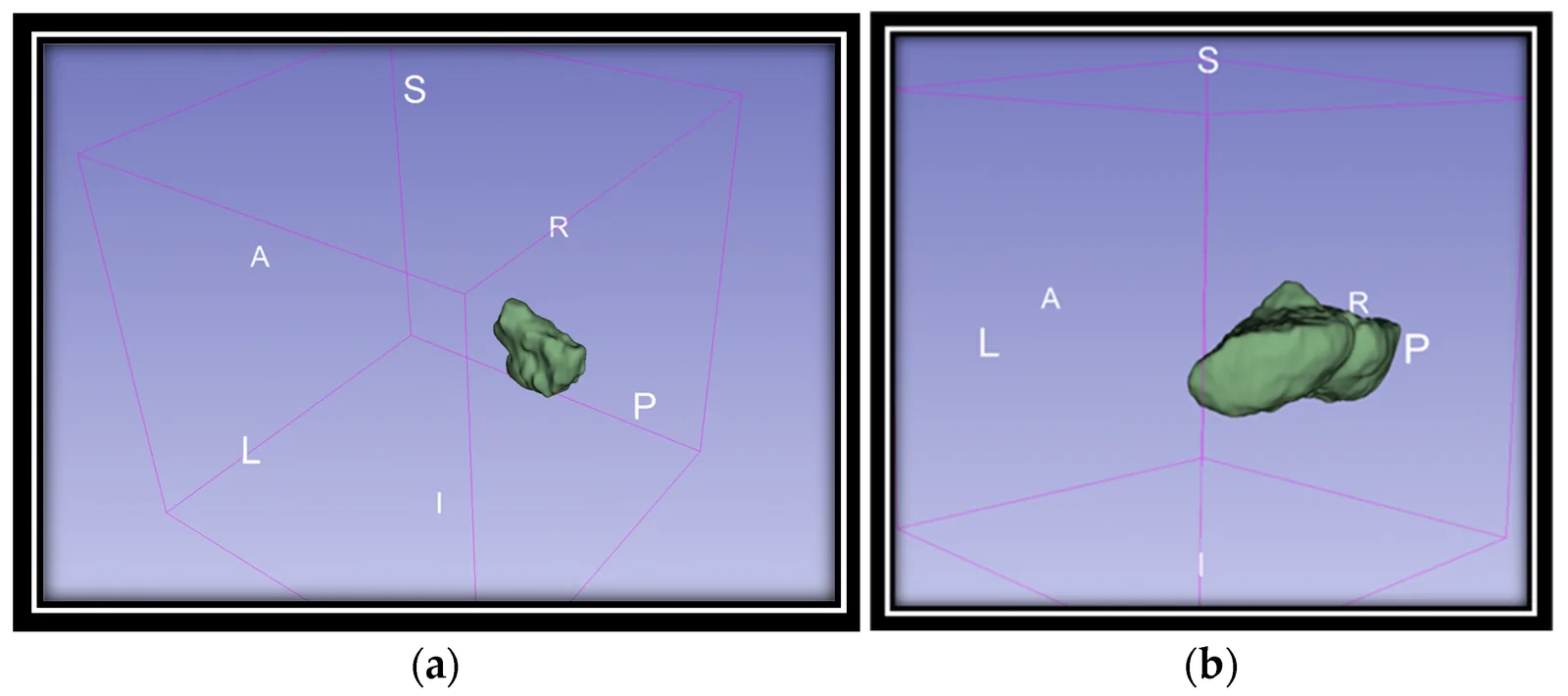
Three-dimensional manual segmentation (3D Slicer) at baseline thin-slice CT showing structural boundaries of the (**a**) cerebellar vermis and (**b**) total cerebellum prior to LAI aripiprazole initiation.

Upon readmission (marking her 10th psychiatric hospitalization), a comprehensive diagnostic workup was initiated. Laboratory investigations included routine hematological and biochemical panels, an endocrine profile, a urine toxicology screen, and a baseline brain Computed Tomography (CT) scan. The urine toxicology panel returned positive for cannabis, confirming a comorbid substance use disorder. Notably, the brain CT scan revealed mild, localized atrophy of the cerebellum and the cerebellar vermis (Figure 1).

### 2.2. Diagnostic Reformulation and Treatment Alignment

The convergence of the patient’s longitudinal clinical history, rapid-cycle affective instability, the emergence of severe high-risk behaviors, and the neuroimaging findings necessitated a fundamental diagnostic re-evaluation. The previous long-standing diagnosis of “Conduct Disorder” was deemed insufficient to account for the episodic nature of the behavioral crises. Consequently, the clinical formulation was revised to Bipolar I Disorder, Current Episode Manic with Psychotic Features (DSM-5 code: 296.44; ICD-10 code: F31.2) and Comorbid Moderate-to-Severe Cannabis Use Disorder (DSM-5 code: 304.30; ICD-10 code: F12.20), developing on a vulnerable neuroanatomical substrate.

The diagnostic revision was rigorously mapped to DSM-5 criteria, requiring a distinct period of abnormally elevated and irritable mood alongside increased goal-directed activity. The patient met Criterion B by exhibiting five distinct core symptoms: pathologically inflated grandiosity, a drastically reduced sleep requirement (<3 h per night without daytime somnolence), pressured speech, flight of ideas, extreme psychomotor agitation, and severe risk-taking behaviors, directly resulting in a total collapse of socio-adaptive functioning. Regarding baseline clinical phenotyping, a formal cranial nerve and motor neurological examination revealed no classic appendicular or gait ataxia, nystagmus, or dysmetria, confirming that the structural lesion did not functionally compromise the anterior motor cerebellum [18]. While standardized neuropsychological testing was unfeasible during the acute manic crisis due to profound agitation, a review of historical pedagogical records revealed a chronic childhood trajectory of severe executive dysfunction, marked selective inattention, and emotional dysregulation, strongly indicating a comorbid, under-the-radar Attention-Deficit/Hyperactivity Disorder (ADHD) phenotype.

Given the history of high psychiatric acuity and extreme non-adherence, the primary therapeutic objective during the 10th hospitalization shifted toward ensuring long-term adherence and achieving neuroanatomical stabilization. Following comprehensive psychoeducation and after obtaining written informed consent from both the legal guardians (parents) and the adolescent patient, an innovative, off-label maintenance protocol was implemented. The patient was transitioned to a second-generation long-acting injectable (LAI) antipsychotic, specifically aripiprazole monohydrate, administered at a monthly dose of 400 mg intramuscularly [19,20]. The rationale for selecting aripiprazole was twofold: first, its LAI formulation eliminated the high risk of infrequent use and erratic compliance typical of adolescent bipolar cohorts [21,22,23]; second, its unique pharmacodynamic profile as a dopamine D2 partial agonist offered potential neuroprotective and neuroplastic benefits to protect the atrophic structures from further volume loss. The pharmacokinetic stability of the 400 mg dose prevents plasma concentration dips that trigger manic relapse.

To address severe residual executive deficits and optimize fronto-cerebellar tuning, oral methylphenidate (10 mg once daily) was carefully introduced as an adjunct. This was initiated only after robust mood stabilization was achieved, utilizing a strict monitoring protocol consisting of twice-weekly clinical mania ratings to preemptively catch any manic switch. Additionally, oral lithium carbonate (300 mg twice daily) was maintained to provide synergistic mood stabilization and anti-suicidal properties, targeting a therapeutic serum range between 0.6 and 0.8 mEq/L.

### 2.3. Neuroimaging Methodology

Brain neuroimaging was performed utilizing a multi-detector row Computed Tomography (MDCT) scanner (GE Healthcare, Chicago, IL, USA; 64-slice Siemens Somatom Definition AS). Helical acquisition scan parameters included a slice thickness of 1.25 mm, a 512 × 512 matrix size, and a dedicated soft-tissue reconstruction algorithm. Volumetric manual segmentation of the total cerebellum and the cerebellar vermis was subsequently executed using the DICOM data on the open-source 3D Slicer platform (version 5.12.1).

While MRI remains the gold standard for volumetric neuroimaging, manual segmentation on thin-slice CT scans offers a viable alternative in emergency psychiatric settings where MRI is unavailable or unfeasible due to patient agitation [24]. To assess measurement reproducibility, manual segmentations were repeated independently by two blinded investigator cohorts, yielding an inter-rater Dice Similarity Coefficient of 0.89 for the total cerebellum and 0.81 for the cerebellar vermis. Given the inherent limitations of CT spatial resolution and partial volume averaging compared to high-resolution MRI, any calculated numerical fluctuations must be interpreted as structural stabilization and boundary estimation thresholds rather than true biological hypertrophy.

### 2.4. 9-Month Follow-Up and Emergence of Neurological Symptoms

**Figure 2:**
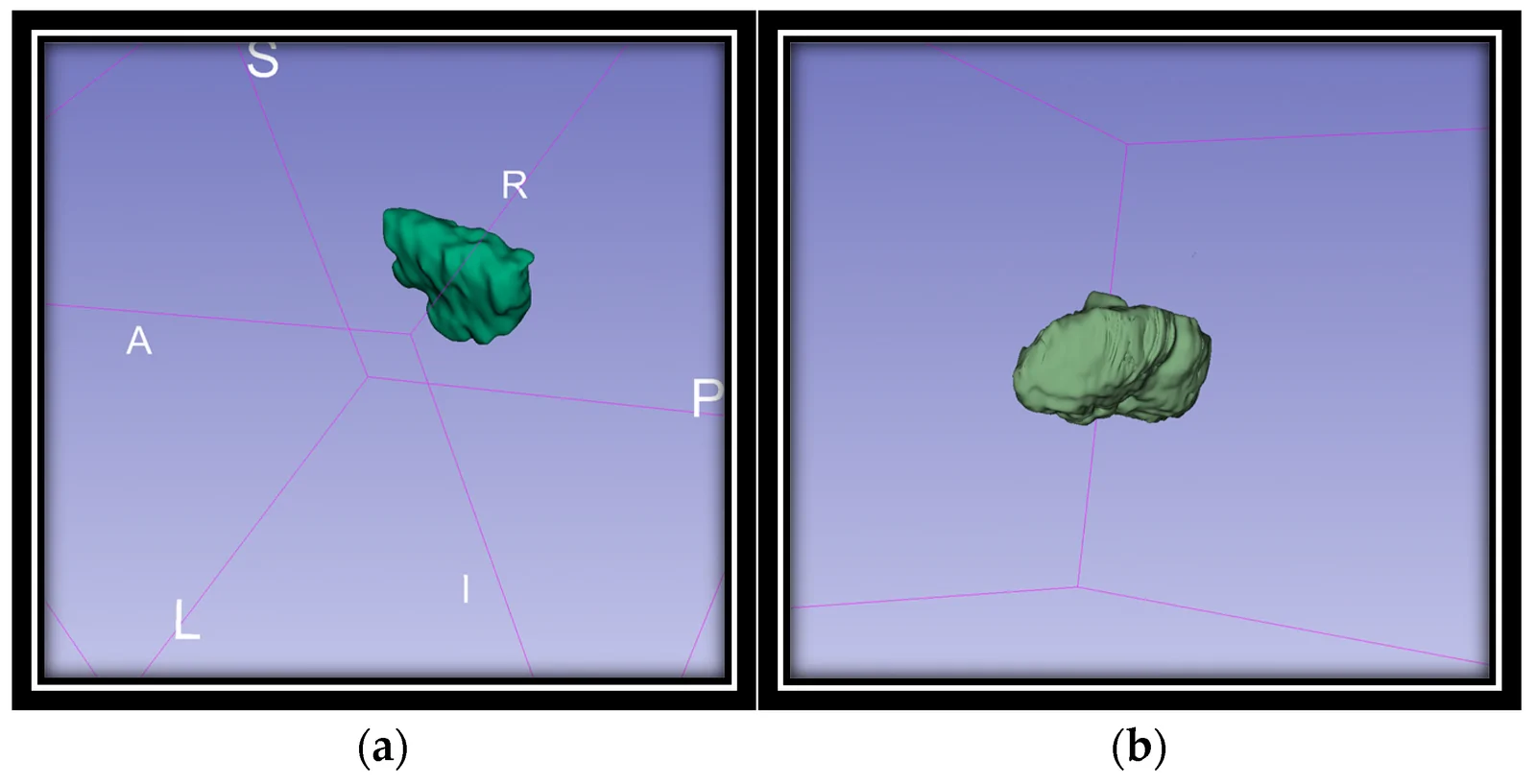
3D manual segmentation (3D Slicer) at 9-month follow-up tracking the (**a**) cerebellar vermis and (**b**) total cerebellum during LAI aripiprazole maintenance.

The patient demonstrated exceptional adherence to the monthly LAI aripiprazole regimen. Clinically, this strategy resulted in a remarkable reversal of the psychiatric illness trajectory, yielding zero psychiatric rehospitalizations or affective relapses. Behaviorally, a profound stabilization of impulse control was achieved, cannabis use remitted completely (confirmed via regular negative urine toxicology screens), and psychosocial adaptation within the family context was highly satisfactory. To monitor the structural deficit, a repeat brain CT scan was performed in the same laboratory by the same senior radiologist at the 9-month mark, suggesting macrostructural stability with no further progression of the atrophy in the cerebellum and vermis (Figure 2; Table 1).

However, during this 9-month stabilization period, the patient experienced an abrupt onset of sudden neurological symptoms. Specifically, at week 38 of maintenance therapy (266 days post-LAI initiation), she experienced an abrupt onset of sudden, daily, brief involuntary muscle jerks localized bilaterally to her upper extremities, occurring predominantly in clusters during morning hours. Due to these acute myoclonic episodes, she was urgently referred to the Neurology Department for further diagnostic evaluation.

An awake electroencephalogram (EEG) was performed, revealing a highly pathological cortical profile. The posterior background activity was measured at 8 Hz, displaying appropriate attenuation upon eye-opening. No drowsiness or Stage Z sleep architecture was captured, and no localized slow-wave activity or focal slowing was registered. Muscle, movement, electrode, and blink artifacts appeared sporadically.

Crucially, the EEG documented intense epileptiform activity against a stable background. The recording captured frequent interictal and ictal epileptiform discharges, characterized by bilateral, synchronous bursts of generalized 3–4 Hz spike-wave complexes lasting 2–4 s, with shifting frontal lead-in and amplitude predominance. These bursts were directly accompanied by clinical correlations of myoclonus during the recording. Hyperventilation significantly exacerbated and increased the frequency of these paroxysmal epileptiform discharges [25,26]. Simultaneous electrocardiography (ECG) confirmed a normal sinus rhythm. The final neurological consensus concluded a highly pathological awake EEG, establishing the presence of diffuse cortical hyperexcitability with a lower seizure threshold.

Following the neurological consensus, a proactive treatment adjustment was implemented: the oral methylphenidate was immediately discontinued, and anti-seizure therapy with levetiracetam (500 mg twice daily) was initiated. The LAI aripiprazole maintenance dose was kept intact due to its critical psychiatric utility. This targeted adjustment resulted in complete clinical remission of the myoclonic episodes within 14 days and subsequent normalization of follow-up EEG profiles.

## 3. Discussion

This case report highlights an intricate, dual neurobiological observation in an adolescent patient undergoing maintenance therapy with LAI aripiprazole for complex BD and cannabis use disorder. While the therapy achieved its primary clinical targets—yielding psychiatric remission and the apparent structural stabilization of the cerebellum and vermis—it simultaneously unmasked a neuroelectrophysiological vulnerability, culminating in cortical hyperexcitability, generalized EEG alterations, and clinical myoclonus. This juxtaposition of macrostructural volume preservation and microstructural electrical destabilization offers valuable insights into the functional topography of fronto-cerebellar networks.

The relationship between structural cerebellar pathology and major psychoses is historically grounded. Heath et al. (1982) [18] demonstrated that approximately 50% of patients with functional psychoses exhibited prominent cerebellar vermal atrophy upon visual inspection of computed tomography (CT) scans, compared to a mere 0.5% to 3.7% in control groups. Contemporary neuroscience has established that the cerebellum plays a critical role in cognitive processing and emotional regulation, a concept heavily implicated in the “cognitive dysmetria” hypothesis [11]. Crucially, the integrity of cerebellar-cortical networks dictates clinical trajectories. Utilizing baseline resting-state functional MRI (rs-fMRI), Velioglu et al. (2025) [12] revealed that stronger baseline functional connectivity between cerebellar cognitive systems and midline cerebral structures predicts superior treatment outcomes across both aripiprazole and risperidone cohorts.

The lack of detectable progression in volumetric changes over 9 months points toward an apparent absence of detectable macrostructural regression during aripiprazole maintenance. Given the substantial methodological limitations and manual segmentation variability acknowledged herein—particularly the calculated +24.77% change in vermis volume—this trajectory is more appropriately described as an absence of detectable change on repeat CT rather than true biological tissue stabilization [24]. Cumulative affective episodes, high-stress states, and chronic cannabis use trigger neurotoxic cascades, including pro-inflammatory cytokine upregulation and accelerated oxidative stress, which may lead to structural gray matter loss [13]. While our clinical data cannot directly measure molecular changes, existing preclinical literature offers hypothetical frameworks that may explain these findings. For instance, the literature suggests aripiprazole may modulate brain-derived neurotrophic factor (BDNF) and attenuate neuroinflammatory pathways in animal models [14,15]; however, these mechanisms remain entirely presumptive in our patient and cannot be verified without fluid biomarkers or advanced functional imaging. Furthermore, it must be emphasized that because no prior childhood neuroimaging was available for comparison, we cannot definitively characterize this baseline cerebellar and vermis atrophy as an active, neuroprogressive degenerative process. It is highly plausible that this structural deficit represents a static, congenital neurodevelopmental variant or a stable hypoplastic vulnerability that primed the patient’s early-onset behavioral phenotype and lowered her baseline threshold for subsequent pharmacological side effects.

The sudden emergence of generalized myoclonus and the highly pathological awake EEG findings—characterized by bursts of generalized 3–4 Hz spike-wave complexes—represent a critical counter-mechanism that warrants rigorous scientific deconstruction. This phenomenon generates the hypothesis of a potential drug-induced lowering of the seizure threshold, which could theoretically be mediated by a complex neuroanatomical mismatch [25,26]. Physiologically, the cerebellum exerts a continuous, modulating inhibitory tone over cerebral cortical structures via the cerebello-thalamo-cortical inhibition (CTCI) pathway. We hypothesize that the patient’s pre-existing, baseline cerebellar and vermis atrophy could theoretically have compromised this homeostatic inhibitory loop, potentially rendering the cerebral cortex electrophysiologically vulnerable. When a high-dose, long-acting atypical antipsychotic was introduced, its complex modulation of serotonin and dopamine receptors—which influence cortical GABAergic interneurons—likely altered cortical polarity and synchronization. In a structurally intact brain, this modification might remain subclinical; however, in this patient’s pre-compromised neuroanatomical substrate, the theorized loss of cerebello-thalamo-cortical inhibition combined with the drug’s electrophysiological footprint unmasked diffuse cortical hyperexcitability.

Crucially, the specific neuroelectrophysiological contribution of LAI aripiprazole cannot be isolated from the patient’s concurrent psychopharmacological regimen. Lithium carbonate possesses well-documented pro-convulsant properties at therapeutic or sub-toxic ranges, frequently inducing intentional tremors, myoclonic jerks, and generalized EEG slowing or paroxysmal discharges when co-administered with atypical antipsychotics. Furthermore, although oral methylphenidate was maintained at a conservative dose (10 mg/day), psychostimulants intrinsically enhance pro-excitatory catecholaminergic neurotransmission. This combination likely created an adverse synergistic effect, where lithium-induced cortical irritability and psychostimulant-mediated excitation converged on a structurally compromised, atrophic cerebellar substrate to precipitate the clinical myoclonus.

A critical differential diagnosis that must be rigorously debated given the patient’s demographic profile is Juvenile Myoclonic Epilepsy (JME). JME typically manifests during mid-to-late adolescence and is characterized pathognomonically by brief myoclonic jerks of the upper extremities, frequently accompanied by generalized 3–4 Hz spike-wave complexes on an awake EEG—identical to the neurophysiological footprint captured in our patient. Given the lack of a baseline EEG, we cannot exclude an underlying, genetically primed JME phenotype. However, the absence of prior childhood absence seizures and the strict temporal alignment with high-dose maintenance therapy strongly favor a drug-induced or polypharmacy-precipitated unmasking of cortical hyperexcitability. This phenotypic intersection highlights the emerging role of the cerebellum in global epilepsy networks. Far from being a purely motor or emotional structure, recent data confirm that the cerebellum acts as a powerful endogenous seizure modulator. Specifically, the cerebello-thalamo-cortical pathway can exert an inhibitory ‘braking’ action on cortical synchronization; we hypothesize that our patient’s baseline cerebellar vermis atrophy structurally compromised this protective brake, allowing the drug-associated electrophysiological footprint to easily precipitate paroxysmal activity. The rapid, total resolution of myoclonus following the addition of levetiracetam (500 mg BID)—a broad-spectrum agent highly effective in both JME and secondary cortical hyperexcitability—further supports this multi-hit network hypothesis.

### Clinical and Research Implications

This case introduces pivotal questions regarding the definition of neuroprotection in clinical psychopharmacology, establishing three distinct dimensions for future research:

1 Structural vs. Electrophysiological Dissociation: Clinicians must recognize that macrostructural volume preservation does not inherently equate to microstructural electrophysiological stability. A therapeutic agent can successfully rescue neuronal populations from inflammation-mediated atrophy via BDNF upregulation while concurrently modifying localized synaptic thresholds to promote paroxysmal activity.
2 The Cerebellum as a Potential Biomarker: Pre-existing cerebellar vermis atrophy or static hypoplasia in adolescent BD may serve as a structural biomarker not only for affective dysregulation but also for heightened vulnerability to drug-induced neuroelectrophysiological adverse events. Patients with documented posterior fossa alterations may require targeted clinical vigilance when exposed to treatments that modulate cortical excitability.
3 Rational Polypharmacy in Dual-Diagnosis Cohorts: The co-administration of LAI antipsychotics and psychostimulants in transitional age youth demands an intricate balancing act. While highly effective in reversing the behavioral illness trajectory and suppressing substance-seeking mechanisms, this combination requires tight clinical vigilance to safely manage the delicate threshold between cognitive optimization and cortical hyperexcitability.

## 4. Limitations

Several significant limitations restrict the generalizability and causal interpretations of this report. First, the lack of a baseline electroencephalogram (EEG) prior to initiating LAI aripiprazole makes it impossible to definitively conclude whether the diffuse cortical hyperexcitability and 3–4 Hz spike-wave complexes developed de novo or represent a pre-existing, subclinical neurodevelopmental trait. Second, neuroimaging was restricted to Computed Tomography (CT) due to acute emergency constraints; thus, manual volumetric boundaries carry a higher margin of measurement error compared to gold-standard MRI, and we cannot definitively exclude subtle parenchymal microstructural abnormalities, focal cortical dysplasias, or deep gray matter lesions outside the posterior fossa that may have independently modulated cortical excitability. Third, because the patient was maintained on rational polypharmacy (including lithium carbonate and methylphenidate), the isolated pharmacodynamic contribution of aripiprazole to both the macrostructural stabilization and the emergent myoclonus cannot be reliably disentangled from potential drug–drug interactions or the standalone neurobiological trajectory of adolescent bipolar disorder.

## 5. Conclusions

In summary, this single clinical observation highlights an intricate neurobiological juxtaposition where apparent macrostructural stabilization of the cerebellum coexisted with a lower seizure threshold in an adolescent patient under complex psychopharmacological maintenance. Due to the inherent limitations of a single-case design, baseline imaging/EEG omissions, and concomitant polypharmacy, no definitive causal relationships can be established, and these findings must be interpreted strictly as an exploratory hypothesis.

This case does not warrant broad changes to clinical guidelines; rather, it underscores the need for larger, prospective, controlled studies utilizing high-resolution MRI and longitudinal neurophysiological tracking to systematically evaluate the safety, boundary errors, and network-level effects of atypical antipsychotics in structurally vulnerable pediatric populations. Consequently, these preliminary findings warrant further prospective clinical exploration. Future research should investigate whether adolescents with documented structural posterior fossa alterations or suspected static hypoplasia might benefit from baseline EEG screening prior to initiating long-acting atypical antipsychotics, followed by systematic longitudinal neurophysiological tracking to clarify the complex network-level equilibrium between macrostructural volume preservation and cortical excitability.

## Figures and Tables

**Table 1:** Clinical, Behavioral, and Neuroanatomical Parameters at Baseline vs. 9-Month Follow-Up.

Parameter	Baseline Assessment (10th Admission)	9-Month Follow-Up (LAI Maintenance)	Volumetric Delta Ratio (Δ)/Clinical Outcome
**Working Diagnosis**	Conduct Disorder (historical attribution)	Bipolar I Disorder (Manic with Psychotic Features)	Confirmed via rigorous DSM-5 mapping
**Adherence Mode**	Oral polypharmacy (high non-compliance)	LAI Aripiprazole monohydrate (400 mg/month)	100% verified compliance trajectory
**Psychiatric Relapses**	9 involuntary admissions within 2 years	Zero (0) readmissions or clinical relapses	Complete psychiatric symptom remission
**Behavioral State**	Severe impulsivity, hypersexuality, elopement	Satisfactory impulse control and stability	Complete behavioral stabilization
**Substance Use**	Active Cannabis Use Disorder (THC positive)	Remission; THC screens consistently negative	Complete cessation of substance use
**Vermis Volume (CT)**	16,014 mm^3^ (59,181 voxels; Surface: 4088 mm^2^)	19,981 mm^3^ (79,919 voxels; Surface: 4510 mm^2^)	Δ = +24.77% * (Methodological boundary error)
**Cerebellar Volume (CT)**	137,328 mm^3^ (507,492 voxels; Surface: 17,359 mm^2^)	139,947 mm^3^ (559,740 voxels; Surface: 17,876 mm^2^)	Δ = +1.90% (Macrostructural stabilization)
**Neurophysiology (EEG)**	Not performed (Major clinical limitation)	Pathological: Bursts of generalized 3–4 Hz spike-wave complexes	Diffuse cortical hyperexcitability and myoclonus

* *Note**:* The high delta ratio (+24.77%) for the vermis volume reflects baseline boundary windowing limits on CT and manual slice selection variability, and should be interpreted strictly as the absence of a detectable change on repeat CT/absence of progression rather than true biological tissue hypertrophy.

## Data Availability

The data used and/or analyzed during the current study are available from the corresponding author on reasonable request.
